# Fuzzy C-means clustering algorithm applied in computed tomography images of patients with intracranial hemorrhage

**DOI:** 10.3389/fninf.2024.1440304

**Published:** 2024-10-23

**Authors:** Lintao Zhang, Dewen Song, Huiying Qiu, Lin Ye, Zengliang Xu

**Affiliations:** ^1^Neurosurgery, Jiaozhou City People’s Hospital, Qingdao, Shandong, China; ^2^Neurosurgery, Jiaozhou City Maternal and Child Health Centre, Qingdao, Shandong, China

**Keywords:** fuzzy C-means clustering (FCM) algorithm, computed tomography (CT) images, intracranial hemorrhage (ICH), convolutional neural network (CNN), image segmentation

## Abstract

In recent years, intracerebral hemorrhage (ICH) has garnered significant attention as a severe cerebrovascular disorder. To enhance the accuracy of ICH detection and segmentation, this study proposed an improved fuzzy C-means (FCM) algorithm and performed a comparative analysis with both traditional FCM and advanced convolutional neural network (CNN) algorithms. Experiments conducted on the publicly available CT-ICH dataset evaluated the performance of these three algorithms in predicting ICH volume. The results demonstrated that the improved FCM algorithm offered notable improvements in computational time and resource consumption compared to the traditional FCM algorithm, while also showing enhanced accuracy. However, it still lagged behind the CNN algorithm in areas such as feature extraction, model generalization, and the ability to handle complex image structures. The study concluded with a discussion of potential directions for further optimizing the FCM algorithm, aiming to bridge the performance gap with CNN algorithms and provide a reference for future research in medical image processing.

## Introduction

Computed tomography (CT) is a non-invasive medical imaging technique (Wang et al., 2021). Because of its low cost, high blood sensitivity, and high efficiency in obtaining results, CT has been the preferred method for the initial diagnosis of intracranial hemorrhage (ICH). Although ICH can be divided into different periods according to the duration of bleeding, the Hounsfield Unit (HU) value of blood is significantly different from that of other brain tissues, which is higher than that of other brain tissues and lower than that of skull (Tamal, 2019). Thus, the main basis for judging the prevalence of ICH is intracranial CT images (Shah et al., 2021). As the scale and volume of medical imaging datasets continue to expand, the role of computer-aided tools in supporting physician diagnosis and treatment has become increasingly critical (Schwenkreis et al., 2021). Traditionally, image segmentation involves dividing an image into non-overlapping regions based on attributes such as intensity or texture (Su et al., 2015; Qiu et al., 2015; Procopio et al., 2022).

Cerebral hemorrhage is the leading cause of death in adults, followed by heart disease and cancer. In most cases, necrosis of bodily organs results from untimely or misdiagnosis of bleeding disorders (Rønning et al., 2021; Martins-Oliveira et al., 2020; Kawata et al., 2017). Timely detection and accurate identification of the location and type of ICH lesions are key factors that directly impact patient survival rates. There are usually five types of cerebral hemorrhage: epidural hemorrhage (EDH), subdural hemorrhage (SDH), subarachnoid hemorrhage (SAH), ICH, and intravascular hemorrhage (IVH). Among them, ICH lesion in the brain tissue was concerned on in this work, because the life of ICH patients is completely dependent on their early diagnosis (Tutunjian et al., 2021; Mine et al., 2021; Lai et al., 2021). At present, foreign scholars have done a lot of research work on CT image segmentation of cranial ICH and formed relatively rich research results, but most of the research results are based on algorithm theory, which is divorced from the clinical application of CT images (Kavitha et al., 2019; Song et al., 2015).

Image segmentation is the process of assigning attributes to each pixel in an image, grouping pixels with similar attributes into distinct regions. This includes dividing digital images into multiple sub-regions to facilitate easier interpretation and analysis (Patel et al., 2021; Ortega Rodriguez et al., 2021; Cao et al., 2021). Segmentation methods are generally categorized into two types: hard segmentation and soft segmentation. Traditional methods fall under hard segmentation, including thresholding, dynamic contours, region growing, and clustering algorithms. In contrast, soft segmentation algorithms incorporate mechanisms to handle uncertainty and ambiguity in image segmentation tasks. Bayesian classification, Fuzzy c-means clustering (FCM) algorithm, and expectation maximization (EM) are soft segmentation methods. Applying these methods to brain CT image segmentation is conductive to distinguishing the brain tissue and cerebrospinal fluid correctly, but brain gray matter and white matter cannot be distinguished (Jalal Deen et al., 2017; Jiang et al., 2018).

In this study, various membership functions were utilized to define the objective using FCM algorithm, and the inflection point of the function was identified as the threshold through optimization of the objective function. Image segmentation can also be viewed as a clustering process. The advantage of the FCM algorithm is its capacity to employ various membership functions for assigning data points to multiple clusters. In this study, the FCM algorithm was enhanced, transitioning from a two-dimensional hybrid algorithm to a three-dimensional one, which was then used to analyze CT images of ICH, with the aim of offering a valuable reference for ICH diagnosis.

## Materials and methods

### Principles for FCM algorithm

The FCM algorithm is a clustering method that evolved from traditional hard clustering techniques. Its core focus is on determining the optimal membership degrees and cluster centers. It was assumed that $M=\left\{m_{1},m_{2},\ldots ,m_{n}\right\}$ was the grayscale value or eigenvalue of the image pixel, f was the number of clusters (number of cluster centers) that divided M, and the cluster center was expressed as $A=\left\{a_{1},a_{2},\ldots ,a_{f}\right\},\mathrm{and}\;b=\left\{b_{xy}\right\}$ was the membership matrix, $b_{xe}$ referred to the degree of membership of $m_{x}$ in e-class area. The cost function expression of FCM was given in Equation 1:


(1) $$\min E_{l}\left(B,A\right)=\overset{F}{\underset{x=1}{\sum }}\overset{n}{\underset{y=1}{\sum }}b_{xy}^{l}r_{xy}^{2}$$

The Equation 2 below had to be satisfied:


(2) $$\left\{ \begin{array}{c} \overset{f}{\underset{x=1}{\sum }}b_{xy}=1,1\leq y\leq n\\ \overset{f}{\underset{y=1}{\sum }}b_{xy}>0,1\leq x\leq f\}\\ b_{xy}=1\leq x\leq f,1\leq e\leq n \end{array}\right.$$

In Equation 2, $B=b_{xy}$ was an n×x fuzzy membership matrix, which represented the size of the membership value of the y-th sample $m_{y}$ belonging to the x-th class, and its range was 0–1; l was the weighted index; $A=\left\{a_{1},a_{2},\ldots ,a_{f}\right\}$ was a h × f matrix composed of f cluster center vectors; and $r_{xy}=||m_{y}-a_{x}||$ meant the Euclidean distance from the sample point $m_{y}$ to the cluster center $a_{x}$ , which was also the 2-norm measurement from the pixel $m_{y}$ to the cluster center.

To minimize the cost function E(B,A), the Lagrange multiplier method was used to establish the objective optimization function, and the objective function was obtained about the cluster center $a_{x}$ , and the partial derivative of the membership degree $b_{xy}$ . In addition, the derivative result was set to zero, and the iterative update expressions for the cluster center and membership degree were obtained as follows:


(3) $$\left\{\begin{array}{c} a_{x}={\left(\overset{f}{\underset{y=1}{\sum }}b_{xy}^{l}\right)}^{-1}\overset{f}{\underset{y=1}{\sum }}{\left(b_{xy}\right)}^{l}m_{y},x=1,2,\ldots ,f\\ T_{y}=\left\{\left(x,y\right)|m_{y}=a_{x},1\leq x\leq f\right\} \end{array}\right.$$

If $T_{y}=\beta$ , the Equation 4 below could be obtained:


(4) $$b_{xy}=\frac{1}{\left[\sum_{g=1}^{f}{r_{gy}}^{-1}r_{xy}\right]},x=1,2,\ldots ,f,y=1,2,\ldots ,n$$

If $T_{y}\neq 0$ , $b_{xy}$ was a nonnegative real number satisfying the below condition, as shown in Equation 5:


(5) $$\overset{f}{\underset{x=1}{\sum }}b_{xe}=1,b_{xe}\in \left[0,1\right]$$

The iterative equation for membership was a mapping from points to sets. In the actual calculation process, the following membership update equation was used:


(6) $$\left\{\begin{array}{c} b_{xy}={\left[\overset{f}{\underset{x=1}{\sum }}{r_{gy}}^{-1}r_{xy}^{\frac{2}{l-1}}\right]}^{-1},T_{y}\neq \beta \\ b_{xy}={\left|T_{y}\right|}^{-1},T_{y}\neq \beta ,x\in T_{y}\\ b_{xy}=0,T_{y}\neq \beta ,x\notin T_{y} \end{array}\}\right.$$

In Equation 6, I represented the number of iterations of the function. If the iteration Equations 3, 7 satisfied the iteration termination condition, that was, i>I or $\max_{x}=||a_{x}^{i+1}-a_{x}^{i}<\alpha ||$ , the iteration should be stopped. After the algorithm completes its iterations, it classifies and segments pixels based on the principle of maximum membership degree. If $b_{yx}>b_{ye}$ , then $m_{y}$ was classified as the x class in cluster center, where e=1,2,…,f;x≠e .

### Improvement of FCM algorithm

The FCM algorithm typically uses Euclidean distance for clustering, which is suitable for clusters with spherical or ellipsoidal distributions. However, when clusters exhibit non-standard shapes, Euclidean distance may fail to capture the relationships between sample dimensions (Okamoto et al., 2021). Therefore, this study introduced a kernel function to measure pixel distances in space. By mapping the lower-dimensional space to a higher-dimensional kernel space, complex nonlinear problems are transformed into linear ones, thereby enhancing the algorithm’s noise resistance. The improvement steps of improving the FCM algorithm were given as follows.

- It should intuitively blurr the image.

The original image was converted from the spatial domain to the fuzzy domain, and grayscale processing was performed on each pixel. For a grayscale image of size C × D, the grayscale level was in the interval $\left[m_{\text{min}},m_{\text{max}}\right]$ , the image was represented by an intuitionistic fuzzy set as follows:


(7) $$Q=\left\{m_{xe},b\left(m_{xe}\right),a\left(m_{xe}\right),\pi \left(m_{xe}\right)\right\},0<x\leq C,0<e\leq D$$

In Equation 7, $b\left(m_{xe}\right)$ was the membership degree of $m_{xe}$ , and $m_{xe}$ was the gray level of the pixel (x,e), which described the degree of brightness and darkness of the gray value of the pixel. The membership and non-membership expressions were shown in Equation 8 and Equation 9, respectively:


(8) $$b\left(m_{xe}\right)={\left(b\left(m_{xe}\right)\right)}^{2}$$

(9) $$a\left(m_{xe}\right)={\left(1-b\left(m_{xe}\right)\right)}^{2}$$

The hesitation of the image after intuition fuzzification was shown in Equation 10:


(10) $$\pi \left(m_{xe}\right)=2b\left(m_{xe}\right)\left(1-b\left(m_{xe}\right)\right)$$

The grayscale value of each pixel was expressed in Equation 11:


(11) $$m_{e}=\left(b\left(m_{xe}\right),a\left(m_{xe}\right),\pi \left(m_{xe}\right)\right)$$

- It can define the initialization parameters.

The membership matrix was extended to the gray level, and the gray level of the image was intuitively fuzzed to obtain the initialized membership matrix *M*. The number of cluster categories was set to *f*, the spatial constraint parameter was *θ*, the weighting index was l, the *δ* referred to the kernel function, the neighborhood radius was *p*, the iteration stop threshold was *α*, the maximum number of iterations was *I*, and the number of initial iterations was 0.

- The local information of pixels was calculated based on their underlying principles.
- The cluster center $A^{*\left(I\right)}=a_{x}^{*\left(I\right)}$ was updated according to Equations 3, 4 as follows, and Equation 12 was obtained:

(12) $$a_{x}^{*\left(I\right)}=\frac{\sum_{y=1}^{n}{\left(b_{xy}^{*\left(I\right)}\right)}^{l}\left(m_{y}+\theta {\bar{m\;}}_{y}\right)}{\sum_{y=1}^{n}\left(1+\theta \right){\left(b_{xy}^{*\left(I\right)}\right)}^{l}}$$

- The membership function matrix $B^{\left|I+1\right|}=b_{xy}^{\left|I+1\right|}$ was updated according to Equation 5 and Equation 13 was obtained:

(13) $$b_{xy}^{\left|I+1\right|}=\overset{f}{\underset{I=1}{\sum }}{\left[\left(\frac{||\varphi \left(m_{y}\right)-\varphi \left(a_{x}\right){\left|\right|}^{2}+\theta ||\varphi \left(m_{y}\right)-\varphi \left({\bar{a\;}}_{x}\right){\left|\right|}^{2}}{||\varphi \left(m_{y}\right)-\varphi \left(a_{p}\right){\left|\right|}^{2}+\theta ||\varphi \left(m_{y}\right)-\varphi \left({\bar{a\;}}_{p}\right){\left|\right|}^{2}}\right)\right]}^{-\frac{1}{l-1}}$$

- The generated hesitation degree $\pi_{xy}^{\left(I+1\right)}$ was adopted to modify the membership degree $B^{\left(I+1\right)}=b_{xy}^{\left(I+1\right)}$ , and Equation 14 and Equation 15 were obtained:

(14) $$b_{ey}^{\left(I+1\right)}=\max\left\{b_{xy}^{\left(I+1\right)}\right\}$$

(15) $$\left\{\begin{array}{c} b_{ey}^{\left(I+1\right)}=1-\pi_{xy}^{\left(I+1\right)}\underset{y\neq e}{\sum }b_{xy}^{\left(I+1\right)}\\ b_{xy}^{\left(I+1\right)}=\pi_{xy}^{\left(I+1\right)}b_{xy}^{\left(I+1\right)} \end{array}\right.\}$$

- Next, it should judge whether the conditions for iterative stop were met $B^{\left(I+1\right)}-B^{I}<\alpha$ . If the conditions were met, the iteration stopped; otherwise, i = i + 1 was defined to repeat the step (e) for the next iteration;
- The image deblurring process involves substituting the membership values corresponding to each grayscale level from the intuitionistic fuzzy partition matrix into the image. This process enables pixel classification based on the principle of maximum membership degree.

### Verification of algorithm segmentation performance

**Figure 1:**
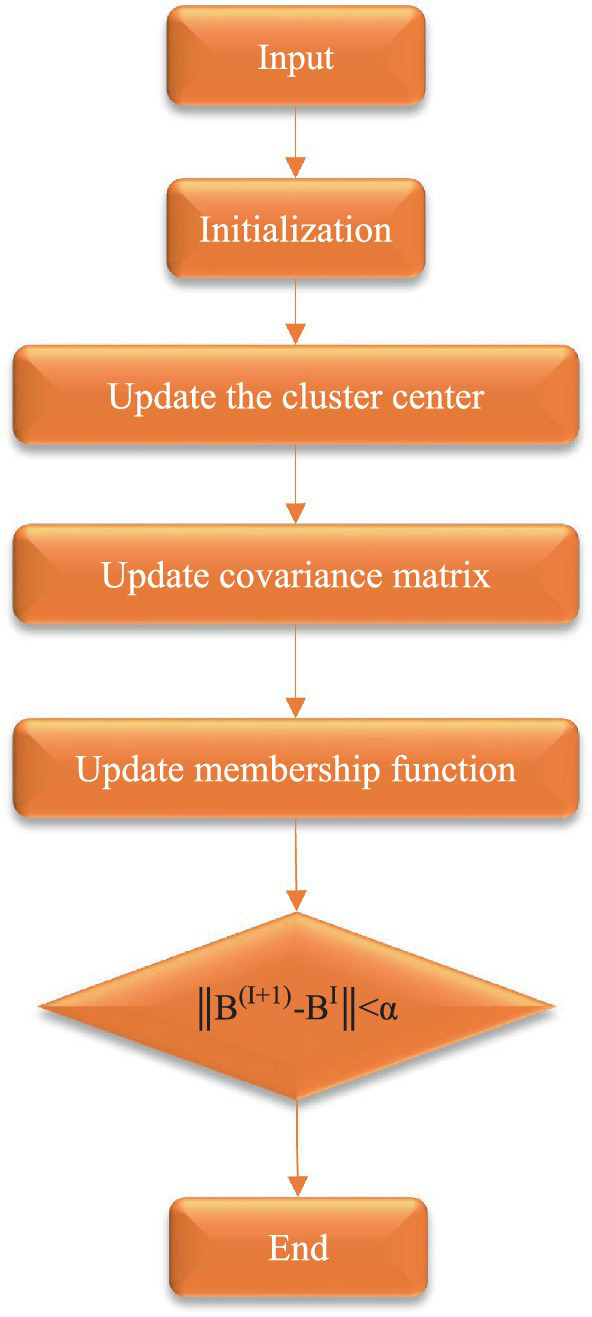
Flow chart for segmentation of algorithm.

Relying solely on human judgment is insufficient for objectively assessing the success of the improved algorithm. Evaluation based on segmentation results provides a more reliable method for assessing algorithm quality. Additionally, the incorporation of various evaluation metrics through quantitative analysis enhances the credibility of the assessment process. To better compare the segmentation performance of different algorithms, three evaluation indicators were introduced for analysis: partition coefficient (Vpc), partition entropy (Vpe), and Xie-Beni index (Vxb). The segmentation performances of the algorithms were shown in Figure 1.

### Preprocessing of CT images

The dataset used in this section was CT-ICH, a public dataset for ICH, which collected CT scans from 113 patients, of which 41 patients had ICH. Each patient had 25 CT slices with a slice thickness of 6 mm. The mean age and standard deviation of age of the patients were 28.2 and 18.7, respectively. Areas with bleeding were marked by specialized radiologists.

For the data format, the original authors of the dataset have windowed images in DICOM (Digital Imaging and Communications in Medicine) format, using two windows: brain (window level = 40 and window width = 120) and skull (window level = 700 and window width = 120) to composite images and save them in NIFTI format.

**Figure 2:**
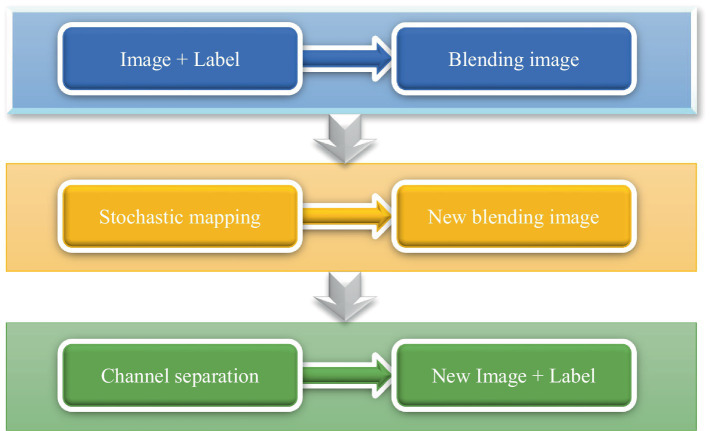
Flow chart of data augmentation.

Given that the dataset includes non-hemorrhagic cases, this study focused on the segmentation of ICH lesions. Consequently, the dataset was reorganized to include only hemorrhagic images and their corresponding annotations, resulting in a total of 256 CT slices. Due to the insufficient number of 256 images for training the proposed network model, data augmentation techniques were employed to expand the dataset. Since the bleeding CT slice had the label of the bleeding area, the CT image and the label should be carried out at the same time during data expansion. The data expansion process was shown in Figure 2.

1. The CT image and the label were channel-fused to obtain a fusion image;
2. The fused images were performed with random rotation transformation, flip transformation, scaling transformation, contrast transformation, noise disturbance, and other data enhancement processing to obtain a new fused image;
3. Channel splitting was applied to the newly fused image to generate a new CT image and corresponding label.

### Experimental setting

Graphic processing unit (GPU) was accelerated training with an NVIDIA RTX 2080TI graphics card. The dataset used in the experimental part was the dataset obtained after data processing. During training, the optimizer used was the Adam optimizer. The initial learning rate was set to 0.0002, and the adjustment strategy of the learning rate was adjusted according to the training loss. If the training loss was constant every 2 epochs, the learning rate was reduced by a factor of 0.6. The resolution of the image was set to 512 * 512, the batchsize was set to 18, and a total of 100 epochs were trained.

### Analysis of performance evaluation metrics

To quantitatively assess the performance of image segmentation, multiple metrics were employed. These metrics specifically include the Dice similarity coefficient, root mean square error (RMSE), and average algorithm runtime. Higher Dice similarity coefficients, along with lower RMSE and average runtime, indicate greater accuracy in image segmentation. The calculations for these metrics are provided in Equations 16, 17.


(16) $$\mathrm{Dice}=\frac{2|E_{i}-R_{i}|}{\left(\left|E_{i}|+|R_{i}\right|\right)}$$

(17) $$\mathrm{RMSE}=\sqrt{\frac{1}{N}}\overset{N}{\underset{i=1}{\sum }}{\left(E_{i}-R_{i}\right)}^{2}$$

In these equations, *Dice*, *E*, *R*, *RMSE*, and *N* represent the Dice similarity coefficient, segmented tissue area, ground truth tissue area, root mean square error, and total number of pixels, respectively.

### Evaluation metrics for lesion volume prediction

During the testing phase, 256 CT images were analyzed and volume predictions were made. The algorithm predicted the hemorrhage probability for each voxel; if the probability exceeded 0.5, the voxel was classified as hemorrhagic, otherwise as normal. In comparing the performance of traditional FCM algorithms, the improved FCM algorithm, and convolutional neural network (CNN) for lesion volume prediction, the objective was to evaluate the differences in their predictive capabilities. CNN, a deep learning algorithm particularly suited for image data, operates on the principle of extracting spatial features through convolutional operations and building abstract feature representations layer by layer. Typically, a CNN comprises multiple convolutional layers for feature extraction, pooling layers for dimensionality reduction and feature compression, and fully connected layers that integrate features to complete classification or regression tasks. Introducing the CNN algorithm into this comparison allows for an effective assessment of the strengths and limitations of each method under varying complexities and conditions, facilitating the selection of the most suitable volume prediction approach for practical applications.

### Computational efficiency and real-time adoption potential of algorithms

To assess the computational efficiency and real-time application potential of FCM, improved FCM, and CNN in clinical settings, the aim was to determine which algorithm was better suited for rapid and accurate processing of ICH or other medical imaging tasks in practical clinical scenarios. A dataset of 256 CT images with varying volumes of ICH was used to simulate diverse conditions within a clinical environment. The three algorithms were executed on standardized hardware (equipped with CPUs of equivalent computational capacity) to ensure fairness in testing. The evaluation metrics are as follows: (1) Computation time: the processing time per image, measured in seconds (s), reflects the algorithm’s computational efficiency. (2) Latency: the total time from image input to result output, measured in seconds (s), indicates the algorithm’s potential for real-time applications. (3) Throughput: the number of images processed per second (frames per second, FPS), used to gage the algorithm’s handling capacity under high load conditions.

(4) Memory usage: the amount of memory occupied by the algorithm during execution, measured in megabytes (MB), reflects the algorithm’s resource consumption.

### Statistical analysis methods

IBM SPSS Statistics 26 was adopted for statistics. The age and disease course of the subjects were expressed as mean ± standard deviation ( $\bar{x\;}$ ± s). The normality test was performed, and the rank sum test was performed for those that did not conform to the normal distribution. The variance homogeneity test was performed for the normal distribution, and the two-sample t test was performed to compare the parameters of the high-level and low-level groups. If the variances were not homogeneous, the corrected t-test was used. In addition, *p* < 0.05 was considered to be statistically significant.

## Results and discussion

### Images using the improved algorithm

**Figure 3:**
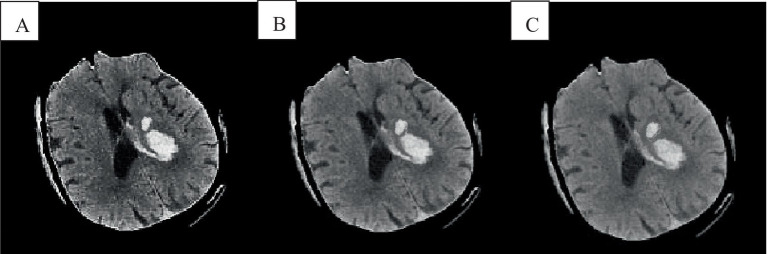
Cranial CT image clustering operation result diagram. **A** was the brain source image, **B** was the result of FCM fuzzy clustering, and **C** was the result processed by the preliminary algorithm.

**Figure 4:**
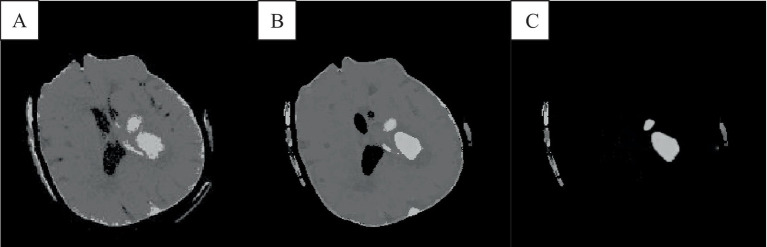
The result of the improved brain CT image clustering operation. **A** was the result of adding the spatial algorithm, **B** showed the result of the improved algorithm, and **C** showed the result of the analysis of the singular point by the improved algorithm.

In the absence of noise interference, the FCM algorithm effectively distinguishes between white matter, gray matter, cerebrospinal fluid, and lesion regions in images. However, due to the high correlation between pixels, adjacent pixels exhibit almost identical data characteristics. This spatial relationship between adjacent pixels plays a crucial role in the segmentation process. General edge detection techniques use this spatial information for image segmentation, but the standard FCM algorithm does not fully utilize this spatial information. In the standard FCM algorithm, abnormal feature data (such as noise) were easily misclassified, such as pseudo-spots of brain gray matter appearing in the white matter of the brain, as shown in Figures 3, 4.

In Figure 3B, the FCM was more sensitive to noise. In the improved FCM algorithm, the membership function of pixels was redefined according to the correlation between pixels. The proximity effect caused the improved FCM algorithm to favor uniformly labeled segments. In Figure 3C, after enhancement of the FCM algorithm, isolated artifacts were reduced, resulting in more uniform image segmentation. A higher parameter *q* enhances the smoothing effect of the improved FCM algorithm, although this may also blur details and reduce segmentation accuracy. When processing noise-contaminated images, the standard FCM algorithm was more prone to confusing white matter with gray matter compared to the improved FCM algorithm. Noise altered the locations of white matter and gray matter pixels, leading to increased misclassification.

The FCM spatial processing algorithm was effective for grayscale images disturbed by noise (Figure 4A). In this algorithm, the median value in the neighborhood centered on the pixel was used to represent spatial information, which meant that the FCM spatial processing algorithm would smooth the image, so although FCM was not sensitive to noise interference, it would cause damage of the images. In this section, the improved algorithm achieved iterative assignment of each pixel to the cluster with the highest membership degree by integrating spatial information into the membership function. Incorporating spatial information into the objective function reduced sensitivity to noise. Images e and f illustrate accurately classified intracranial structures in the absence of noise, with clearer differentiation between white matter and gray matter. The algorithm also maintained accurate classification in the presence of noise, owing to its dual consideration of spatial information and enhanced noise resistance, thereby preserving image details.

### Segmentation results of lesions in brain CT images

**Figure 5:**
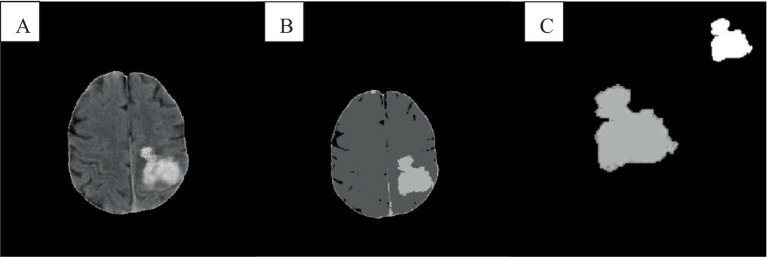
Comparison of lesion segmentation of cranial CT. **A** was the intracranial CT image, **B** showed the FCM clustering result, and **C** showed the segmentation result of the algorithm.

The experimental results were shown in Figure 5, where the size of each image was 512 × 512. In this group of experiments, a total of 2 algorithms were used, namely the standard FCM and the algorithm in this work. Next, a comparative analysis of the two algorithms was conducted using specific parameters: *l* was set to 2, alpha = 0.0001, and the neighborhood size was 5 × 5. After the intracranial CT images were classified with the clustering algorithms, the segmented images were further processed using a region-growing algorithm to highlight the significant differences between the two algorithms.

### Comparison on lesion area

**Figure 6:**
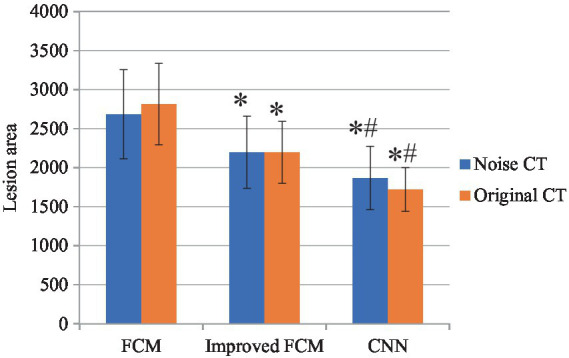
Comparison of lesion area. “*” indicated compared to FCM group, *p* < 0.05; “#” indicated compared to improved FCM group, *p* < 0.05.

In Figure 6, among noisy CT images, the CNN algorithm exhibited the lowest prediction error, indicating its superior capability in handling data and providing more accurate volume predictions in the presence of noise. The improved FCM algorithm followed, showing an enhancement over the traditional FCM algorithm. The traditional FCM algorithm demonstrated the highest error, reflecting its poorest performance on noisy images. In the case of original CT images, the CNN algorithm also achieved the lowest prediction error, demonstrating its continued excellence in noise-free conditions. The improved FCM algorithm performed slightly worse than CNN on original images but still outperformed the traditional FCM algorithm. The traditional FCM algorithm’s error remained the highest, indicating its inferior performance compared to the other two algorithms, even in the absence of noise.

### Comparison on FCM algorithm analysis difference

**Figure 7:**
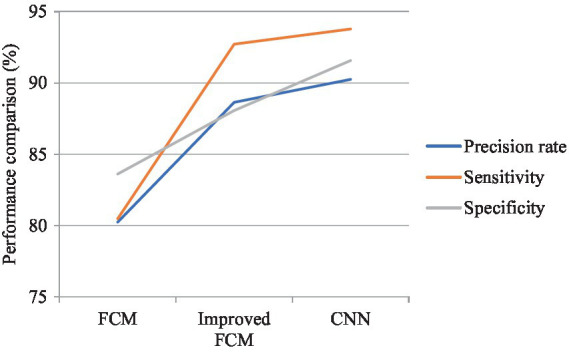
Comparison on difference between three algorithms.

In Figure 7, the performance of the three algorithms was compared across three metrics: precision rate, sensitivity, and specificity. The CNN algorithm demonstrated the highest precision rate, indicating its superior ability to accurately identify true lesion regions and minimize misclassification. The improved FCM algorithm also showed a significant increase in precision, approximately 8% higher than that of the traditional FCM algorithm. The traditional FCM algorithm exhibited the lowest precision rate, suggesting a higher tendency for errors in prediction. The CNN algorithm achieved the highest sensitivity, reflecting its exceptional capability in detecting actual lesions. The improved FCM algorithm also showed a considerable improvement in sensitivity, approaching that of the CNN algorithm. The traditional FCM algorithm had the lowest sensitivity, indicating a higher likelihood of missing some lesion areas. Regarding specificity, the CNN algorithm performed best, demonstrating its accuracy in distinguishing non-lesion regions and reducing false positives in normal tissue. The improved FCM algorithm also showed an enhancement in specificity, allowing for better differentiation of non-lesion areas. The traditional FCM algorithm had relatively lower specificity, implying a higher rate of false positives.

### Graphical abstract

**Figure 8:**
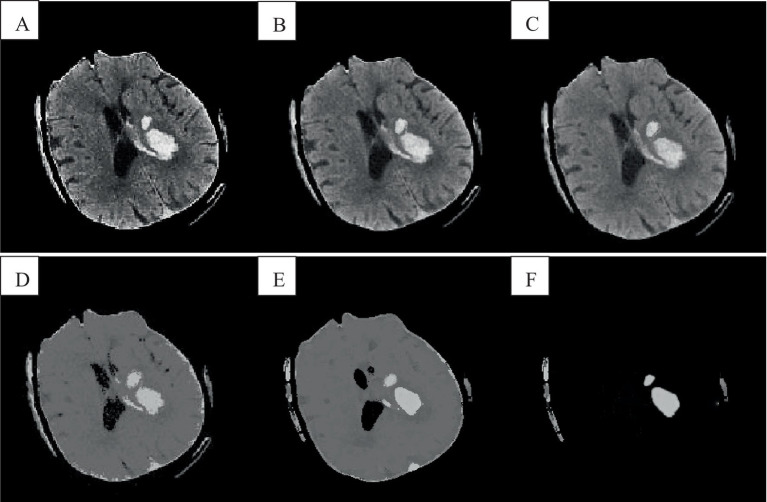
Cranial CT image clustering operation result diagram. **A** was the brain source image, **B** was the result of FCM fuzzy clustering, and **C** was the result processed by the preliminary algorithm, **D** was the result of adding the spatial algorithm, **E** showed the result of the improved algorithm, and **F** showed the result of the analysis of the singular point by the improved algorithm.

The FCM spatial processing algorithm was effective for grayscale images disturbed by noise (Figure 8D). In this algorithm, the median value in the neighborhood centered on the pixel was used to represent spatial information, which meant that the FCM spatial processing algorithm would smooth the image, so although FCM was not sensitive to noise interference, it would cause damage of the images. The improved algorithm in this section allowed each pixel to be assigned to the cluster with the highest membership in an iterative process by incorporating spatial information into the membership function. By incorporating spatial information into the objective function, the sensitivity to noise can be reduced. Observing images e and f revealed that intracranial images can be accurately classified without noise interference, resulting in clearer differentiation between brain white matter and gray matter. Correct classification was also possible in the presence of noise interference. Because of the consideration of dual spatial information, the algorithm was insensitive to noise and destroys the details of the image less.

### Comparison of segmentation performance across different algorithms

In Table 1, the CNN algorithm achieved the highest Dice similarity coefficient of 0.93, indicating the greatest overlap with the ground truth in segmentation tasks and thus the highest segmentation accuracy. The improved FCM algorithm followed, with a Dice similarity coefficient of 0.91, representing a significant improvement over the traditional FCM algorithm’s coefficient of 0.85. The traditional FCM algorithm had the lowest Dice coefficient, reflecting its relatively poorer segmentation performance and lower overlap with the ground truth. The CNN algorithm also had the lowest RMSE of 0.095, demonstrating the smallest segmentation error and minimal deviation from the ground truth. The improved FCM algorithm ranked next, with an RMSE of 0.097, showing a notable reduction compared to the traditional FCM algorithm’s RMSE of 0.117, indicating an improvement in precision with the enhanced FCM approach. The traditional FCM algorithm had the highest RMSE, signifying a larger segmentation error and greater deviation from the ground truth. In terms of average processing time, the CNN algorithm performed the fastest with an average time of 101 s, reflecting both high accuracy and efficiency. The improved FCM algorithm had a slightly longer average processing time of 107 s, but this was an improvement over the traditional FCM algorithm. The traditional FCM algorithm had the longest average processing time of 146 s, indicating not only lower accuracy but also reduced efficiency in segmentation tasks.

**Table 1:** Segmentation performance of different algorithms.

Algorithm	Dice similarity coefficient	Root mean square error	Average time (s)
FCM	0.85	0.117	146
Improved FCM	0.91	0.097	107
CNN	0.93	0.095	101

### Comparison of ICH volume between two algorithms

In Table 2, for predicting ICH volumes less than 5 mL, the CNN algorithm had the smallest absolute error (0.49 ± 0.35 mL), indicating the highest prediction accuracy for small-volume hemorrhages. The improved FCM algorithm slightly lagged behind the CNN, while the traditional FCM algorithm exhibited the largest error. In terms of relative error, the traditional FCM algorithm performed slightly better than the improved FCM and CNN algorithms, although the differences among the three were minor and their overall performances were relatively close. For medium-volume hemorrhages (5–25 mL), the CNN algorithm had the lowest absolute error (1.45 ± 1.16 mL) and relative error (0.15 ± 0.05%), indicating the highest prediction accuracy within this volume range. The improved FCM algorithm followed, showing significant improvement over the traditional FCM algorithm. The traditional FCM algorithm had the highest errors, particularly in absolute error, indicating weaker performance in predicting medium-volume hemorrhages. For large-volume hemorrhages (>25 mL), the improved FCM algorithm had the smallest absolute error (7.56 ± 5.19 mL), slightly better than the CNN algorithm (7.61 ± 4.72 mL), while the traditional FCM algorithm had a significantly larger error (18.26 ± 12.71 mL), reflecting the poorest performance. Regarding relative error, the improved FCM algorithm had the lowest error (0.10 ± 0.08%), followed closely by the CNN algorithm, with the traditional FCM algorithm exhibiting the highest relative error.

**Table 2:** Comparison of ICH volume between two algorithms.

ICH volume	Absolute error in hemorrhage volume (mL) FCM Improved FCM CNN	Relative error in hemorrhage volume (%) FCM Improved FCM CNN
<5 mL (*n* = 70)	0.64 ± 0.34 0.51 ± 0.46 0.49 ± 0.35	0.31 ± 0.15 0.35 ± 0.37 0.34 ± 0.27
5–25 mL (*n* = 102)	2.67 ± 1.84 1.48 ± 1.34 1.45 ± 1.16	0.24 ± 0.13 0.17 ± 0.06 0.15 ± 0.05
>25 mL (*n* = 84)	18.26 ± 12.71 7.56 ± 5.19 7.61 ± 4.72	0.24 ± 0.16 0.10 ± 0.08 0.12 ± 0.06

### Comparative analysis of algorithm applicability and advantages

In a clinical environment, shorter computation times enable physicians to obtain results more rapidly. In Table 3, the CNN algorithm had the shortest computation time (1.01 s per image), indicating its advantage in efficiency. Shorter latency times reflect higher potential for real-time application. The CNN algorithm also had the shortest latency time (1.05 s), followed closely by the improved FCM algorithm (1.12 s), both demonstrating strong real-time application capabilities. Throughput measures the algorithm’s processing capability under high load conditions, with the CNN algorithm exhibiting the highest throughput (0.95 frames per second, FPS), making it suitable for clinical scenarios requiring rapid processing of large volumes of images. Lower memory usage indicates reduced resource consumption by the algorithm. Although the CNN algorithm exceled in computational efficiency, it had a higher memory usage (640 MB), which may be less suitable in resource-constrained environments compared to the FCM algorithm (524 MB).

**Table 3:** Comparative analysis of algorithm applicability and advantages.

Index	FCM	Improved FCM	CNN
Computation Time (s/image)	1.46	1.07	1.01
Latency (s)	1.50	1.12	1.05
Throughput (FPS)	0.68	0.93	0.95
Memory usage (MB)	524	618	640

## Discussion

In recent years, with the development and popularization of artificial intelligence technology, its influence has penetrated into all walks of life, and the medical field is no exception. Imaging technology in medicine is a necessary means for the early diagnosis and evaluation of many major diseases. However, many data and the lack of human experience have led to the inability to confirm and treat the disease in a timely manner (Turcato et al., 2021; Shalaby et al., 2021; Rundo et al., 2020; Podolsky-Gondim et al., 2021). ICH is one of the major diseases that threaten human health. CT is a commonly used imaging technique for ICH, and different ICH types have different characteristics. Presenting different states in different modes is a tedious and time-consuming task for direct manual segmentation of CT images. At present, many researchers have done a lot of research and made some progress, but there is still a certain gap from clinical application. For this reason, the FCM algorithm was improved, and on this basis, the segmentation of CT images provided reference to the research on the diagnostic value of ICH (Munarriz et al., 2021).

The results of clustering aligned with the objectives of image segmentation, leading to the widespread use of clustering algorithms in this field. In real life, the probability of the existence of fuzzy events was much greater than that of precise events, thus resulting in fuzzy clustering algorithms (Kang et al., 2021; Jasim and Brindha, 2021; Huang et al., 2020). FCM algorithm is one of the classical algorithms of fuzzy clustering, but FCM algorithm still has defects in many aspects. Many scholars have devoted themselves to the study of improved FCM algorithms, among which the representative ones are IFCM algorithm and SFCM algorithm (Cheswick et al., 2019; Callejas-Moraga et al., 2020; Cajander et al., 2016). In this work, the improved FCM was analyzed deeply, aiming to find a more objective and effective improvement method.

Based on the segmentation performance of different algorithms, our experiments yielded the following results: The Dice similarity coefficient for the improved FCM algorithm reached 0.91, surpassing the 0.85 achieved by the traditional FCM algorithm, indicating a significant improvement in segmentation accuracy. The RMSE of the improved FCM algorithm was 0.097, lower than the 0.117 of the traditional FCM algorithm, further demonstrating smaller and more precise segmentation errors. The average processing time for the improved FCM algorithm was 107 s, notably shorter than the 146 s required by the traditional FCM algorithm, highlighting the improvement in computational efficiency. In terms of performance, the improved FCM algorithm showed significant advancements in both Dice similarity coefficient and average processing time. This indicates that the improved FCM algorithm not only achieved more accurate image segmentation but also completed computations more rapidly, which is crucial for large-scale medical image processing. Across all volume ranges, the improved FCM algorithm consistently exhibited lower absolute errors compared to the traditional FCM algorithm, with a particularly significant reduction in absolute errors for larger volumes (>25 mL). This indicated that the improved algorithm could accurately predict hemorrhage volumes in practical applications. For volumes <5 mL, although the improved FCM algorithm showed smaller absolute errors, its relative error was slightly higher than that of the traditional FCM algorithm. This sensitivity in relative error could be attributed to the inherently smaller error range for small hemorrhage volumes. By enhancing both the accuracy and efficiency of image segmentation, the improved FCM algorithm was able to better assist clinicians in diagnosing and treating ICH, thereby improving the efficiency and accuracy of clinical workflows.

CNN is a prototypical deep learning algorithm that, unlike traditional clustering-based FCM algorithms, can automatically extract more advanced and complex features. Therefore, comparing the performance of CNN with that of FCM and improved FCM algorithms allows for the assessment of whether deep learning methods offer significant advantages in handling medical imaging tasks such as ICH volume prediction and image segmentation. Through this comparison, a more rigorous performance evaluation of the improved FCM algorithm can be conducted. If the improved FCM algorithm performs comparably to or better than the CNN algorithm on certain metrics, it would validate the effectiveness of these enhancements. Conversely, if the CNN algorithm significantly outperforms the improved FCM algorithm, it may indicate that deep learning methods are more suitable for handling complex imaging tasks. Additionally, this comparison provides insights into the relative strengths and weaknesses of the three algorithms for various clinical applications. For example, while CNN may offer superior accuracy, it requires higher computational resources; in contrast, FCM algorithms may be easier to implement and consume fewer resources. By comparing performance, researchers can select the most appropriate algorithm based on specific clinical needs. The experimental results indicate that the CNN algorithm exhibited the lowest errors when processing both types of CT images, demonstrating strong adaptability and accuracy, particularly in handling complex or noisy images. The improved FCM algorithm showed moderate performance on noisy and original CT images, with some improvement over the traditional FCM algorithm but still lagged behind the CNN algorithm. The traditional FCM algorithm had higher errors in both types of images, especially in noisy CT images, reflecting relatively poorer performance. This suggests that the CNN algorithm has a significant advantage in volume prediction tasks, particularly in processing noisy CT images, where its robustness and predictive accuracy outperform both the traditional and improved FCM algorithms.

Based on the experimental results presented in this study, the CNN algorithm outperformed on all three performance metrics—precision, sensitivity, and specificity—demonstrating the best overall performance in lesion volume prediction. It not only achieved higher accuracy in detecting and predicting lesion areas but also excelled in minimizing false positives. The improved FCM algorithm showed significant advancements over the traditional FCM algorithm, particularly in sensitivity and precision, approaching the performance of the CNN algorithm. However, it still fell short of the CNN in terms of specificity. The traditional FCM algorithm performed poorly across all metrics, indicating a tendency for both missed detections of lesions and false positives in normal tissue. The introduction of the CNN algorithm substantially enhanced the accuracy and reliability of lesion volume predictions, underscoring its higher clinical value for practical applications. The improved FCM algorithm also demonstrated good performance but did not reach the level of the CNN algorithm. The traditional FCM algorithm, on the other hand, showed clear limitations in this task. The CNN algorithm excelled in segmentation performance, achieving the highest Dice similarity coefficient and the lowest RMSE, while also processing images at the fastest speed, highlighting its superiority in image segmentation tasks. Although the improved FCM algorithm showed significant improvement over the traditional FCM in terms of Dice similarity coefficient and RMSE, and also had reduced processing time, it still fell short compared to the CNN algorithm. The traditional FCM algorithm underperformed across all three metrics, particularly in processing time, indicating deficiencies in both efficiency and accuracy for practical applications. In image segmentation tasks, the CNN algorithm stands out as the best choice due to its higher accuracy and efficiency. The improved FCM algorithm offers enhanced performance but still lags behind the CNN algorithm. The traditional FCM algorithm remains relatively weaker across all metrics and is best suited for situations with less demanding requirements or limited resources.

In predicting small and medium volumes of ICH, the CNN algorithm demonstrated the best performance, with the lowest absolute and relative errors, indicating its strongest predictive capability within these volume ranges. The improved FCM algorithm slightly outperformed the CNN algorithm in predicting large volumes of hemorrhage but showed somewhat lower performance compared to CNN for small and medium volumes. Overall, the improved FCM algorithm represents a significant enhancement over the traditional FCM algorithm. The traditional FCM algorithm exhibited weak performance across all volume ranges, particularly with significantly larger errors in predicting large-volume hemorrhages, highlighting its inefficacy in handling complex or large-volume hemorrhages. For predicting hemorrhage volume, the CNN algorithm has a clear advantage for small and medium volumes, while the improved FCM algorithm shows slight superiority for large volumes. The traditional FCM algorithm performed relatively poorly, with prediction accuracy across all volume ranges falling short of the other two algorithms. In terms of computational efficiency and accuracy, the CNN algorithm also performs exceptionally well, making it particularly suitable for clinical environments with high real-time requirements. However, it does have a drawback in memory usage. The improved FCM algorithm, while more efficient than the traditional FCM algorithm, maintains a relatively balanced memory usage, making it suitable for scenarios with limited resources but requiring a certain level of real-time performance. Although the traditional FCM algorithm has lower memory usage, it underperforms in computation time and throughput, making it more appropriate for clinical tasks where real-time performance is less critical. These evaluations enable more targeted algorithm selection in clinical environments, aligning with the specific needs of different application scenarios.

By comparing with advanced CNN algorithms, it can be analyzed that the improved FCM algorithm proposed in this study still requires further enhancement in several aspects and exhibits some gaps relative to the CNN algorithm. The CNN algorithm possesses powerful feature extraction capabilities, enabling it to automatically extract multi-layered, high-dimensional features from images. In contrast, both the traditional FCM and the improved FCM algorithms primarily rely on manually designed features and clustering strategies. This disparity results in the CNN algorithm achieving higher accuracy in handling complex image tasks, particularly in capturing subtle structures and intricate shapes. To further enhance the feature extraction capabilities of the FCM algorithm, incorporating more sophisticated feature extraction methods or integrating some feature extraction layers from deep learning models to create a hybrid model could address these limitations. Due to its deep learning architecture, the CNN algorithm demonstrates strong generalization ability, maintaining high performance across different image datasets. In contrast, the FCM algorithm and its improved version may exhibit less stability when confronted with variations in datasets or noise interference. To enhance the generalization capability of the improved FCM algorithm, strategies such as data augmentation, regularization methods, and the integration of other types of learning algorithms can be employed to improve model performance across various datasets. The CNN algorithm is particularly effective at handling complex structures in images, such as edges and textures, and performs exceptionally well in processing multimodal medical images. In contrast, the improved FCM algorithm may rely on parameter tuning and empirical rules when dealing with complex image structures, demonstrating less flexibility and adaptability. To address these limitations, adaptive parameter adjustment mechanisms could be introduced into the FCM algorithm, or multi-scale analysis methods could be integrated to enhance its capability in processing complex image structures. Although the improved FCM algorithm might outperform CNN in terms of computational efficiency and resource consumption, it may reveal shortcomings under high-precision requirements, especially during large-scale data processing. CNN’s advantage in parallel computing allows it to handle vast amounts of data more efficiently without significant loss of accuracy. To bridge this gap, efforts could be made to optimize the computational workflow of the FCM algorithm by incorporating parallel computing techniques or GPU acceleration, while preserving its resource efficiency. Additionally, exploring the design of lightweight models could further improve computational efficiency while maintaining accuracy.

In other research, Balakrishnan et al. focused primarily on the early detection and diagnosis of cardiovascular diseases, particularly leveraging medical image processing techniques (Balakrishnan and Ambeth Kumar, 2023). The study utilized Internet of Things (IoT)-based devices to collect patient health records and echocardiogram images. The research involved preprocessing and segmenting these images, followed by classification and cardiac risk prediction using deep learning techniques. Specifically, image segmentation was performed using the FCM algorithm, while classification employed a pre-trained recurrent neural network (PRCNN). The results demonstrated that the proposed method achieved an accuracy of 99.5%, surpassing current state-of-the-art technologies. This underscores the extensive application of clustering algorithms, particularly FCM, in medical image segmentation. However, with advancements in medical imaging technology and increasing data complexity, traditional clustering algorithms face several challenges, including sensitivity to noise, high computational complexity, and limited ability to handle complex structural shapes. Therefore, researchers have sought to enhance these algorithms through various approaches to improve their application effectiveness in medical imaging. Scholars have improved traditional clustering algorithms by integrating spatial information of pixels, enabling them to handle noise and local inconsistencies in medical images more effectively. This approach can enhance both the accuracy and robustness of segmentation. Additionally, combining clustering algorithms with deep learning techniques can further improve medical image segmentation performance. For instance, integrating FCM with CNN to create a hybrid model can leverage CNN’s feature extraction capabilities to enhance the segmentation performance of the clustering algorithm.

The study by Liao et al. primarily focused on analyzing ultrasound images of Duchenne muscular dystrophy (DMD) patients using deep learning and clustering algorithms (Liao et al., 2024), aiming to predict patients’ mobility and assess the severity of the disease. The research employed k-means and FCM clustering algorithms to reconstruct texture features of the DMD dataset, and various classification models were used to evaluate the accuracy of disease stage and mobility function recognition. The results demonstrated that deep CNNs VGG-16 and VGG-19 achieved an accuracy of 98.53% in classifying mobility function, with VGG-19 achieving an accuracy of 92.64% in classifying disease severity. The study indicated that reconstructing texture features with clustering algorithms, combined with machine learning and deep learning techniques, can effectively assist in identifying DMD symptoms and tracking disease progression. Similarly, our study can explore the application of clustering algorithms to the segmentation of ICH lesions in CT images by reconstructing texture features of the CT images to enhance the clarity and separability of the lesion regions. This approach may assist in more accurately identifying and segmenting ICH lesions, particularly in cases where boundaries are unclear or noise levels are high. The study can draw upon these ideas by attempting to integrate various features of CT images—such as density, texture, and shape—using clustering algorithms to classify and aggregate different features, thereby improving the segmentation of ICH lesions. Additionally, it may be beneficial to introduce deep learning models to refine the preliminary segmentation results based on the improved FCM algorithm, further enhancing the accuracy and robustness of the segmentation. The combination of deep learning and clustering algorithms could provide a more powerful tool for the automatic segmentation of ICH lesions.

It is important to note that in practical applications, the sources and quality of medical images may vary. Therefore, when the methods proposed in this study are applied to new clinical scenarios, additional tuning and validation may be required to ensure the algorithm continues to perform well on new data. This limitation serves as a reminder to researchers and clinicians to carefully evaluate the algorithm based on specific application contexts to avoid misjudgments or misdiagnoses due to insufficient generalization ability. In addition to using a broader dataset, researchers could explore cross-domain validation or test image data from different hospitals and equipment. This approach helps to verify the robustness and generalization capability of the algorithm, ensuring its reliability under different imaging conditions. Additionally, the complexity of the proposed algorithm implies that its implementation in actual clinical settings may face challenges. Clinical personnel may need a high level of expertise and technical background to properly understand and apply the algorithm. Additionally, implementing the algorithm may require additional computational resources and technical support, such as high-performance computing devices or specialized software environments, which could pose a barrier for some medical institutions. Furthermore, this study primarily focused on improving and enhancing the algorithm’s performance rather than extensive clinical validation. Although the algorithm demonstrated good results on the utilized dataset, there is a lack of broad clinical validation across diverse patient datasets. This means that the applicability of the research findings to other clinical scenarios or different patient types remains uncertain, potentially limiting the credibility of widespread application. Future plans include developing user-friendly software tools or interfaces that encapsulate the complex algorithm into straightforward operational workflows, allowing clinical personnel to use the tool without needing a deep understanding of the technical details. Detailed technical guidelines and training resources will be provided to help clinical technicians acquire the necessary knowledge and skills. Exploration of optimized versions of the algorithm will aim to reduce computational resource requirements, enabling it to operate on standard medical equipment. Concurrently, multi-center studies should be conducted, utilizing datasets from different hospitals and diverse patient populations to perform extensive clinical validation of the algorithm. Collaboration with clinicians and radiology experts will be essential to evaluate the algorithm’s performance in real clinical environments and make improvements based on feedback. Expanding the algorithm’s application to more disease types or imaging modalities will be crucial for validating its generalizability and reliability across different clinical adoptions.

## Conclusion and suggestions

### Conclusion

This work primarily focused on the segmentation of brain CT images and proposed a comprehensive and effective segmentation method. However, it lacked detailed processing and did not include a thorough data comparison. Future research could explore additional aspects and integrate insights from various fields. Regarding the follow-up research work of the paper, it intended to improve the segmentation theory in this work to further improve the accuracy of the segmentation results. To enhance the neighborhood spatial information, adjustment factors can be introduced for optimization, taking into account pixel correlations. Additionally, combining complementary segmentation methods can improve the algorithm’s efficiency and accuracy. The improved FCM algorithm and its hemorrhage region segmentation technique successfully achieved the separation and delineation of unclear and incomplete hemorrhage edges in CT images. This approach significantly enhanced noise resistance and accelerated the segmentation process.

Based on a comprehensive review of existing literature and research findings, this study focused on developing a computer-aided diagnostic system for CT images. The system encompasses the segmentation of hemorrhage regions and the quantification of hematoma volumes. These advancements assist clinicians in rapidly and accurately assessing patient conditions, particularly in determining hematoma volumes, thereby facilitating the formulation of effective treatment plans. This study holds significant practical value for clinical applications.

### Innovations and advantages

This work mainly introduced the whole process from ICH CT original image to final ICH lesion segmentation. First, the DICOM file was converted into a BMP image. Then, the images were preprocessed. To reduce the interference of other tissues such as the skull on the extraction of the lesion area, the intracranial structure extraction operation was performed. Finally, the intracranial structure was clustered, and the ICH lesion was extracted by the region growing algorithm on the classified images.

This work proposed two improvements: first, addressing the issue of identical boundary weights selected by the LOG operator during image preprocessing; and second, incorporating a boundary intensity detector into the LOG operator to measure the intensity of selected edge points at each zero-crossing. By employing the enhanced LOG operator for edge extraction in this study, false edge points can be effectively filtered out, resulting in clear and accurate edge delineation.

The second improvement point was that the FCM algorithm did not consider the spatial information of pixels in the clustering process to improve. A significant improvement in this study is the incorporation of spatial information into the definitions of the membership function and the objective function. This method can perform accurate and efficient segmentation of ICH lesions without prior manual intervention, and was insensitive to noise.

According to the ICH lesion segmentation workflow proposed in this study, ICH CT images were processed. Experimental results indicated that the algorithm can accurately segment ICH lesions even when the hemorrhage regions are adjacent to surrounding tissues. The algorithm demonstrated effective segmentation and can offer essential technical support for the subsequent measurement of bleeding volume, thereby exhibiting notable clinical value.

### Suggestions

This study has several areas that require further improvement. Firstly, the analysis primarily focused on traditional image processing algorithms within the context of algorithm research. Looking ahead, future research aims to integrate lesion segmentation with machine learning methods to enhance both the speed and accuracy of the segmentation process. Second, learning three-dimensional (3D) reconstruction technology can reconstruct the extracted lesions in 3D, which is more helpful for the quantification of lesion volume, diagnosis, and pathological localization research.

Medical image segmentation plays a vital role in many medical imaging applications. The methods for medical image segmentation are primarily categorized into automatic and semi-automatic approaches. In future study and research, we should focus on researching automated algorithm segmentation to make the processing of medical images more accurate and intelligent.

## Data Availability

The original contributions presented in the study are included in the article/supplementary material, further inquiries can be directed to the corresponding authors.
